# Author Correction: A universal language for finding mass spectrometry data patterns

**DOI:** 10.1038/s41592-025-02785-1

**Published:** 2025-08-08

**Authors:** Tito Damiani, Alan K. Jarmusch, Allegra T. Aron, Daniel Petras, Vanessa V. Phelan, Haoqi Nina Zhao, Wout Bittremieux, Deepa D. Acharya, Mohammed M. A. Ahmed, Anelize Bauermeister, Matthew J. Bertin, Paul D. Boudreau, Ricardo M. Borges, Benjamin P. Bowen, Christopher J. Brown, Fernanda O. Chagas, Kenneth D. Clevenger, Mario S. P. Correia, William J. Crandall, Max Crüsemann, Eoin Fahy, Oliver Fiehn, Neha Garg, William H. Gerwick, Jeffrey R. Gilbert, Daniel Globisch, Paulo Wender P. Gomes, Steffen Heuckeroth, C. Andrew James, Scott A. Jarmusch, Sarvar A. Kakhkhorov, Kyo Bin Kang, Nikolas Kessler, Roland D. Kersten, Hyunwoo Kim, Riley D. Kirk, Oliver Kohlbacher, Eftychia E. Kontou, Ken Liu, Itzel Lizama-Chamu, Gordon T. Luu, Tal Luzzatto Knaan, Helena Mannochio-Russo, Michael T. Marty, Yuki Matsuzawa, Andrew C. McAvoy, Laura-Isobel McCall, Osama G. Mohamed, Omri Nahor, Heiko Neuweger, Timo H. J. Niedermeyer, Kozo Nishida, Trent R. Northen, Kirsten E. Overdahl, Johannes Rainer, Raphael Reher, Elys Rodriguez, Timo T. Sachsenberg, Laura M. Sanchez, Robin Schmid, Cole Stevens, Shankar Subramaniam, Zhenyu Tian, Ashootosh Tripathi, Hiroshi Tsugawa, Justin J. J. van der Hooft, Andrea Vicini, Axel Walter, Tilmann Weber, Quanbo Xiong, Tao Xu, Tomáš Pluskal, Pieter C. Dorrestein, Mingxun Wang

**Affiliations:** 1 Institute of Organic Chemistry and Biochemistry of the Czech Academy of Sciences, Prague, Czech Republic; 2 Metabolomics Core Facility, Immunity, Inflammation, and Disease Laboratory, Division of Intramural Research, National Institute of Environmental Health Sciences, National Institutes of Health, Research Triangle Park, NC USA; 3 Department of Chemistry and Biochemistry, University of Denver, Denver, CO USA; 4 Functional Metabolomics Lab, CMFI Cluster of Excellence, University of Tuebingen, Tuebingen, Germany; 5 Department of Biochemistry, University of California Riverside, Riverside, CA USA; 6 Department of Pharmaceutical Sciences, Skaggs School of Pharmacy and Pharmaceutical Sciences, University of Colorado Anschutz Medical Campus, Aurora, CO USA; 7 Collaborative Mass Spectrometry Innovation Center, Skaggs School of Pharmacy and Pharmaceutical Sciences, University of California San Diego, La Jolla, CA USA; 8 Biologicals and Natural Products Discovery, Crop Protection R&D, Corteva Agrisciences, Indianapolis, IN USA; 9 BioMolecular Sciences, School of Pharmacy, University of Mississippi, Oxford, MS USA; 10 Pharmacognosy, Faculty of Pharmacy, Al-Azhar University, Nasr City, Egypt; 11 Department of Fundamental Chemistry, Institute of Chemistry, University of São Paulo, São Paulo, Brazil; 12 Department of Chemistry, Case Western Reserve University, Cleveland, OH USA; 13 Walter Mors Institute of Research on Natural Products, Federal University of Rio de Janeiro, Rio de Janeiro, Brazil; 14 Environmental Genomics and Systems Biology Division, Lawrence Berkeley National Lab, Berkeley, CA USA; 15 The Joint Genome Institute, Lawrence Berkeley National Lab, Berkeley, CA USA; 16 Mass Spectrometry Center of Expertise, Regulatory and Stewardship, Corteva Agrisciences, Indianapolis, IN USA; 17 Biologicals and Natural Products, Crop Protection R&D, Corteva Agrisciences, Indianapolis, IN USA; 18 Department of Chemistry – BMC, Science for Life Laboratory, Uppsala University, Uppsala, Sweden; 19 Clinical Biomarkers Laboratory, School of Medicine, Emory University, Atlanta, GA USA; 20 Institute of Pharmaceutical Biology, University of Bonn, Bonn, Germany; 21 Institute of Pharmaceutical Biology, Goethe University Frankfurt, Frankfurt, Germany; 22 Department of Bioengineering, University of California San Diego, La Jolla, CA USA; 23 West Coast Metabolomics Center, University of California Davis, Davis, CA USA; 24 School of Chemistry and Biochemistry, Center for Microbial Dynamics and Infection, Georgia Institute of Technology, Atlanta, GA USA; 25 Scripps Institution of Oceanography and Skaggs School of Pharmacy and Pharmaceutical Sciences, University of California San Diego, La Jolla, CA USA; 26 Faculty of Chemistry, Institute of Exact and Natural Science, Federal University of Para, Belem, Brazil; 27 Institute of Inorganic and Analytical Chemistry, University of Münster, Münster, Germany; 28 Center for Urban Waters, University of Washington, Tacoma, WA USA; 29 Department of Biotechnology and Biomedicine, Technical University of Denmark, Kongens Lyngby, Denmark; 30 Laboratory of Physical and Chemical Methods of Research, Center for Advanced Technologies, Tashkent, Uzbekistan; 31 College of Pharmacy, Sookmyung Women’s University, Seoul, Republic of Korea; 32 SW R&D Bioinformatics, Life Science Mass Spectrometry, Bruker Daltonics GmbH & Co. KG, Bremen, Germany; 33 Department of Medicinal Chemistry, College of Pharmacy, University of Michigan, Ann Arbor, MI USA; 34 College of Pharmacy and Integrated Research Institute for Drug Development, Dongguk University-Seoul, Goyang, Republic of Korea; 35 College of Pharmacy, University of Rhode Island, Kingston, RI USA; 36 Applied Bioinformatics, Department of Computer Science, University of Tuebingen, University of Tuebingen; Institute for Bioinformatics and Medical Informatics, University of Tuebingen; Institute for Translational Bioinformatics, University Hospital Tuebingen, Tübingen, Germany; 37 The Novo Nordisk Foundation Center for Biosustainability, Technical University of Denmark, Kongens Lyngby, Denmark; 38 Department of Chemistry and Biochemistry, UC Santa Cruz, Santa Cruz, CA USA; 39 Department of Marine Biology, The Leon H. Charney School of Marine Sciences, University of Haifa, Haifa, Israel; 40 Department of Chemistry and Biochemistry, University of Arizona, Tucson, AZ USA; 41 Department of Biotechnology and Life Science, Tokyo University of Agriculture and Technology, Koganei, Japan; 42 School of Chemistry and Biochemistry, Georgia Institute of Technology, Atlanta, GA USA; 43 Department of Chemistry and Biochemistry, San Diego State University, San Diego, CA USA; 44 Pharmacognosy Department, Faculty of Pharmacy, Cairo University, Cairo, Egypt; 45 Natural Products Discovery Core, Life Sciences Institute, University of Michigan, Ann Arbor, MI USA; 46 Institute of Pharmacy, Freie Universität Berlin, Berlin, Germany; 47 Institute for Biomedicine, Eurac Research, Bolzano, Italy; 48 Department of Pharmacy, University of Marburg, Marburg, Germany; 49 Applied Bioinformatics, Department of Computer Science, University of Tuebingen, University of Tuebingen, Tübingen, Germany; 50 Department of BioMolecular Sciences, School of Pharmacy, University of Mississippi, Oxford, MS USA; 51 Chemistry and Chemical Biology, Northeastern University, Boston, MA USA; 52 RIKEN Center for Integrative Medical Sciences, Tsurumi-ku, Japan; 53 RIKEN Center for Sustainable Resource Science, Tsurumi-ku, Japan; 54 Bioinformatics Group, Wageningen University & Research, Wageningen, the Netherlands; 55 Department of Biochemistry, University of Johannesburg, Johannesburg, South Africa; 56 Crop Protection R&D, Corteva Agrisciences, Indianapolis, IN USA; 57 Data Science and Bioinformatics, Corteva Agrisciences, Dublin, OH USA; 58 Department of Computer Science, University of California Riverside, Riverside, CA USA

**Keywords:** Computational platforms and environments, Metabolomics

Correction to: *Nature Methods* 10.1038/s41592-025-02660-z, published online 12 May 2025.

This article was originally published under standard Springer Nature license (© The Author(s), under exclusive licence to Springer Nature America, Inc.). It is now available as an open-access paper under a Creative Commons Attribution 4.0 International license, © The Author(s). The error has been corrected in the HTML and PDF versions of the article.

